# Biochemical and Nutritional Changes during Food Processing and Storage

**DOI:** 10.3390/foods8100494

**Published:** 2019-10-14

**Authors:** Vibeke Orlien, Tomas Bolumar

**Affiliations:** 1 Department of Food Science, University of Copenhagen, Rolighedsvej 26, DK-1958 Frederiksberg, Denmark; 2 Department of Safety and Quality of Meat, Max Rubner Institute, Federal Research Institute of Nutrition and Food; E.-C.-Baumann- Straße 20, 95326 Kulmbach, Germany; tomas.bolumar@mri.bund.de

## 1. Introduction

Domestic food processing goes a long way back in time, for example, heat for cooking was used 1.9 million years ago. Nowadays, food and meal preparation seems to be moving out of the home kitchen into factories, and pre-processed or processed/convenience foods are becoming a larger part of the daily diet. In addition, consumers are progressively focusing on the impact of their food on health, and demand foods that have a high nutritional quality, and aroma and natural flavor similar to freshly-made products. Therefore, nutritional quality is concurrent with food safety, and sensory perception is becoming an increasingly important factor in food choices. The human digestive tract disintegrates food in order for nutrients to be released and be made available to the body. However, nutrients can undergo unwanted degradation upon processing and subsequent storage, negatively influencing the nutritional value of food and its physiological effects. Different processing techniques will result in different food structures, thereby also affecting bioaccessibility, bioavailability, and overall nutritional value. Hence, food scientists and industry have an increased interest in both conventional and innovative processing methods that can provide products with good quality and high nutritional value, along with a stable shelf life.

This Special Issue aims to shed some light on the latest knowledge and developments regarding the effects of food processing and storage on biochemical and nutritional changes.

## 2. Effect of Processing and Storage on Biochemical and Nutritional Changes

The processing of food raw material often targets specific compounds in order to achieve the desired texture and taste, for example, treatment of milk in order to coagulate proteins to form a cheese and subsequent storage to develop flavor. In complex matrices such as meat and vegetables, the processing will most likely also affect other compounds and hence produce biochemical changes that may affect the product properties in a negative manner. In meats, the formation of unwanted substances or safety concerns are sometimes related to processing conditions, for instance, the formation of polycyclic aromatic hydrocarbons during intensive particular smoking processes, the generation of heterocyclic aromatic amines in particular heating/ grilling conditions, and the release of compounds from the oxidation reactions of lipids and proteins that can affect flavour and texture [1,2]. The opposite is also possible, and one can favor processes that boost the presence of nutritional valuable components in meat products and/or facilitate digestion. For instance, through an extensive proteolysis, the formation of bioactive peptides with different bioactivity such as antioxidant, antihypertensive, immunomodulating, antimicrobial, prebiotic, and hypocholesterolemic properties can be enhanced in a variety of fermented and aged meat products [3]. The application of mild preservation processes can better preserve highly regarded nutrients such as vitamins.

Obviously, the product goal can be reached without extensive detrimental effects by selecting proper processing parameters. However, this is not a straightforward task, and more knowledge at the molecular level is still needed. This Special Issue covers a total of six articles (five research papers and one commentary report) concerning the effect of new processing technologies, packaging methods, protein extraction, and color ingredients on products or compounds related to biochemical and nutritional changes.

Juices are a well-known healthy food product, produced and enjoyed for centuries, but the juicing process is in fact allowing contact between degradative enzymes and healthy phenolic compounds leading to flavor and nutritional changes. The paper ‘Influence of Pulsed Electric Field and Ohmic Heating Pretreatments on Enzyme and Antioxidant Activity of Fruit and Vegetable Juices’ provides new and valuable information about the optimization of the pulsed electric field (PEF) and ohmic heating (OH) treatments for reducing the energy requirements and process time and increasing yield and quality. Thus, color, antioxidant activity (DPPH and ABTS method), and enzyme (peroxidase and polyphenoloxidase) activity were investigated in carrot and apple juices subjected to various PEF and OH treatments. In conclusion, both PEF and OH were found to positively contribute to improved juice quality by enhanced ingredient release and retention. Fish is also a well-known healthy food, but also highly perishable, and upon storage, protein oxidation and degradation have severe negative effects on nutritional value. The paper ‘Effect of Different Packaging Methods on Protein Oxidation and Degradation of Grouper (Epinephelus coioides) During Refrigerated Storage’ shows how different packaging methods such as air packaging (AP), vacuum packaging (VP), and modified atmosphere packaging (MAP) affect protein oxidation and degradation of grouper fillets during refrigerated storage. By monitoring changes in total sulfhydryl and disulfide bonds, carbonyl content and hydrophobicity, ATPase activity, soluble peptides, myofibril fragmentation index, free amino acids, protein secondary structure, and total protein electrophoresis, the authors showed that the degree of grouper fillet protein oxidation was increased upon storage. Moreover, it was found that protein oxidation and degradation were highly correlated. In conclusion, the high-carbon-dioxide MAP packaging method played a positive role in the inhibition of myofibril degradation and oxidation for refrigerated grouper fillets. Likewise, it is now well-known, for instance, that the use of rich oxygen MAP in other muscle foods such as red meat accelerates oxidative processes that affect color, flavor, and texture. Recent research has found that by application of proper antioxidant strategies and reducing the oxygen concentration in the package, it is possible to mitigate this problem and obtain a product of superior quality at the point of sale [4,5]. The two papers published in this special Issue are examples of how the investigation of mechanism at a molecular level can contribute to an overall assessment of applied technology and packaging on product quality and nutritional value. Both papers are generic in its methodology, which can be transferred to other raw food materials.

Color is an important quality attribute of food products for consumer acceptability. In this issue, two papers provide information about the stability of two natural colorants, namely carotenoids and anthocyanins, which both also have health promoting properties. The paper ‘Stabilization of Crystalline Carotenoids in Carrot Concentrate Powders: Effects of Drying Technology, Carrier Material, and Antioxidants’ seeks to stabilize carrot carotenoid crystals by spray- and freeze-drying, addition of functional additives, and oxygen free storage. An analytical approach was applied in order to qualitatively assess the physical state and the pigment concentration during production and storage. In conclusion, the exclusion of oxygen clearly had the most profound effect on carotenoid stability during storage. On the other hand, in the paper ‘The Influence of Chemical Structure and the Presence of Ascorbic Acid on Anthocyanins Stability and Spectral Properties in Purified Model Systems’ the influence of anthocyanins’ structure, pH, and ascorbic acid on the stability and spectral properties of anthocyanins during simulated shelf life was investigated by spectral and high performance liquid chromatography-mass spectrometry analyses. The systematic stability study showed a higher stability in acidic medium and enhanced stability with increasing size of conjugated sugar, and a rapid and high anthocyanin degradation when stored without cooling and with the addition of ascorbic acid, which both should be avoided to protect anthocyanins from degradation.

The growing population has put a focus on the question of whether there is enough protein to feed the increasing number of humans and animals. Therefore, the examination of protein raw materials is of both scientific and applied interest. The paper ‘Variations in Amino Acid and Protein Profiles in White versus Brown Teff (Eragrostis Tef) Seeds, and Effect of Extraction Methods on Protein Yields’ compares the nutritional qualities by amino acid composition among six teff seed types and three different protein extraction methods. Maybe not surprisingly, a clear genetic variability between white and brown teff seed types was established. Interestingly, brown teff had higher content of essential amino acid than the white type. Moreover, the extraction method gave different results concerning the type of protein extracted and can thus be used to tune the quality or functional differences among teff protein fractions or meals.

The understanding of the digestibility of different foods can also play a decisive role in providing recommendations for nutritional guidelines and how to make the best use of the available food resources. The review paper ‘The Effect of Processing on Digestion of Legume Proteins’ highlights that protein digestibility increases after processing using different processing methods. However, since both the type of legume and the applied methods differed, it cannot be concluded which specific method is best for each individual legume type. Therefore, further research is required at the legume type level to provide processing recommendations that maximize bioavailability.

Biochemical and nutritional changes during food processing and storage have important implications for both consumer protection and health as well as food quality. We forecast many more studies coming up to address the optimization of processing conditions, possibly with the incorporation of novel mild processing methods and packaging processes as a way for food authorities and industry to minimize the presence of unwanted compounds and maximize the quality and nutritional value of food.

We would like to acknowledge all the authors and reviewers for their excellent contribution, effort, and comments.
